# Associations of exposure to blood heavy metal mixtures with *Toxoplasma* infection among U.S. adults: a cross-sectional study

**DOI:** 10.3389/fpubh.2024.1463190

**Published:** 2024-11-19

**Authors:** Jing Zhou, Chen Xing, Yuting Chen, Jilu Shen

**Affiliations:** ^1^Department of Clinical Laboratory, The First Affiliated Hospital of Anhui Medical University, Hefei, Anhui, China; ^2^Department of Clinical Laboratory, Anhui Public Health Clinical Center, Hefei, Anhui, China; ^3^Department of Microbiology, School of Basic Medical The Key Laboratory of Microbiology and Parasitology of Anhui Province, The Key Laboratory of Zoonoses of High Institutions in Anhui, Anhui Medical University, Hefei, Anhui, China; ^4^Department of Epidemiology and Biostatistics, School of Public Health, Anhui Medical University, Hefei, Anhui, China

**Keywords:** *Toxoplasma* infection, heavy metal, mixtures, NHANES, independent and comprehensive associations

## Abstract

**Introduction:**

Research increasingly links environmental exposure to toxic metals with health risks, yet the effect of combined metal exposure on *Toxoplasma* infection remains underexplored. This study investigates the relationship between concurrent heavy metal exposure and *Toxoplasma* infection in adults.

**Methods:**

We analyzed data from 10,746 adults aged 20–80 from NHANES, with 1,869 positive for *Toxoplasma gondii* IgG. The study assessed associations between lead (Pb), cadmium (Cd), and mercury (Hg) with *Toxoplasma* infection risk using single-metal logistic regression, RCS analysis, WQS regression, and qgcomp models.

**Results:**

Each metal showed an independent association with *Toxoplasma* infection risk. Pb had a non-linear association, while Hg had a linear one. Analysis of multiple metals indicated a positive correlation between heavy metal exposure and infection risk, particularly in younger and middle-aged adults, with Pb showing the strongest link.

**Discussion:**

Our findings reveal a significant association between heavy metal exposure and *Toxoplasma* infection risk, especially in younger demographics, with lead being a key factor. This highlights the importance of understanding environmental metal exposure’s impact on public health and informs the development of prevention strategies.

## Introduction

*Toxoplasma gondii*, a single-celled eukaryotic protozoan parasite, is primarily transmitted through oral, bloodborne, and congenital routes of infection. It is estimated that approximately one-third of the global population may be infected with *Toxoplasma gondii* (1, 2). Although *Toxoplasma gondii* infection rates continue to decline in the United States, overall infection rates remain at approximately 7% (3). Individuals experiencing an acute *Toxoplasma* infection may exhibit symptoms akin to the flu. Fortunately, those with robust immune systems can typically eliminate the majority of the parasites during the acute phase. The parasites that survive tend to linger within the body, forming slow-growing cysts composed of bradyzoites, particularly in tissues that are less accessible to immune surveillance, such as the brain, eyes, heart, and skeletal muscles (4). For the majority of adults, the infection does not lead to serious illness. However, congenital infections in children can result in developmental issues such as mental retardation and blindness. Additionally, children with compromised immunity may experience more serious disorders (5).

With the development of industrialization, heavy metals are persistently threatening human health in a series of ways, including industrial products, food, soil, air, and drinking water (6). Environmental pollutants have many negative effects on human health through their diversification, accumulation, and ecological destruction (7). Heavy metals are regarded as a constant threat to humans since they cannot be eradicated (8). Studies have shown that combined exposure to multiple heavy metals can damage innate and adaptive immune responses, leading to enhanced susceptibility to infection and the occurrence of inflammatory and autoimmune disorders (9, 10). Prenatal exposure to heavy metals affects infectious disease-related genes within the glucocorticoid receptor signaling pathways (11). While the immunotoxic effects of heavy metals have been explored through animal studies, *in vitro* experiments, clinical assessments, and occupational exposure studies, there is a scarcity of large-scale human epidemiological research examining the detrimental immunological impacts of exposure to these metals. Furthermore, heavy metals are often found together in the environment, potentially leading to a range of interactions such as additive, antagonistic, synergistic, or other complex effects (12, 13). Hence, there is a need to explore the combined effect of heavy metals on common chronic infections.

Thus far, the relationship between heavy metal exposure and *Toxoplasma* infection has not been well-established. Previous studies on the origin of blood *Toxoplasma* and its interaction with heavy metals were not adequately reviewed. In this research, we leveraged data from the National Health and Nutrition Examination Survey (NHANES), which pertains to the general U.S. population, to conduct a comprehensive assessment of the association between three prevalent blood heavy metals and their combined effects on *Toxoplasma* infection. We used multivariable logistic regression, restricted cubic spline (RCS) analysis, logistic and weighted quantile sum (WQS) regression, and quantile-based g computation (qgcomp) models to elucidate these relationships. The findings of this study are expected to yield novel perspectives for the prevention and management of *Toxoplasma* infections.

## Materials and methods

### Data source and study population

**Figure 1:**
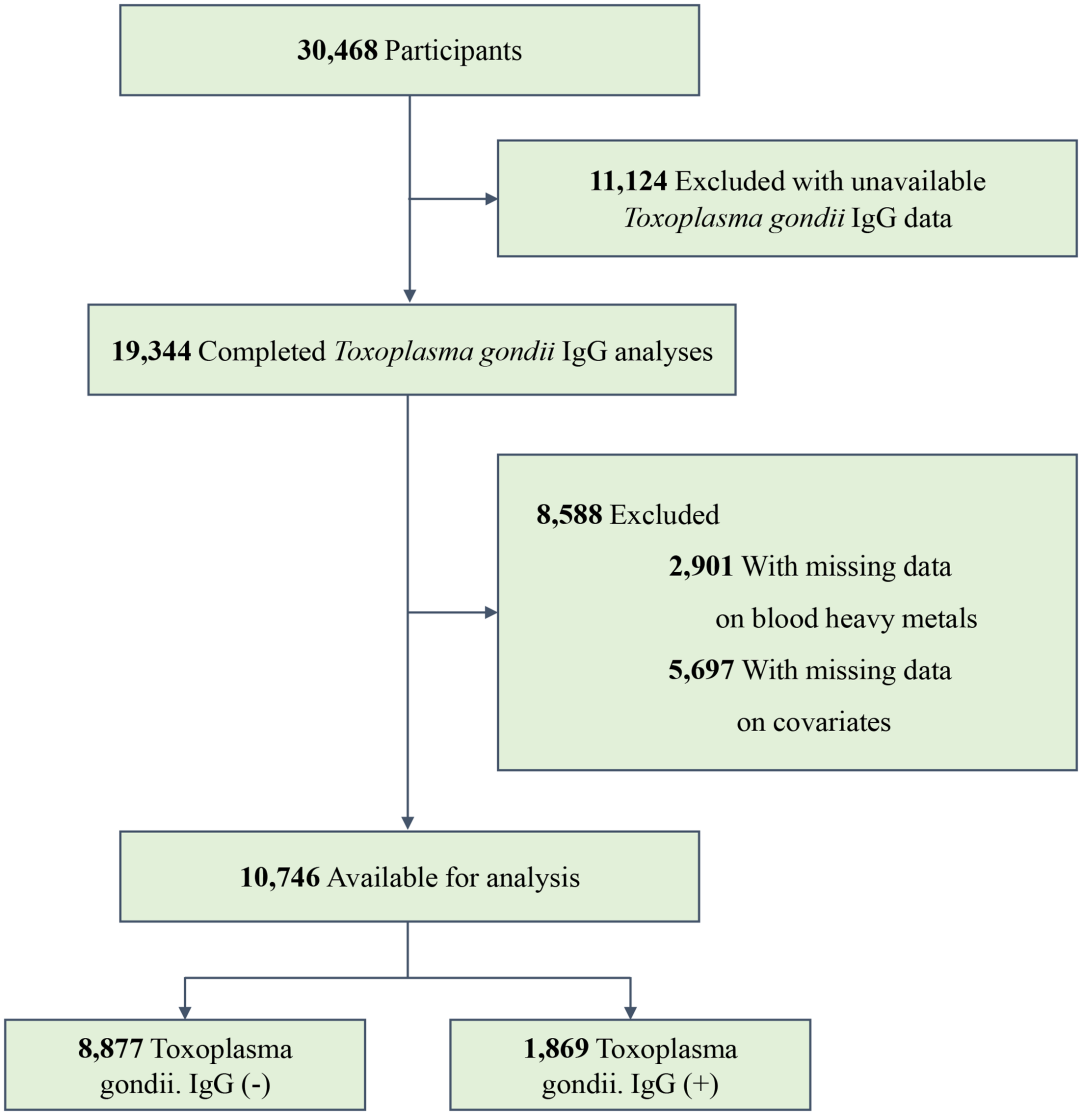
Flow diagram of the screening and enrollment of study participants.

The NHANES is a continual, national, multi-stage, and cross-sectional program conducted by the National Cancer Research Center for Health Statistics (NCHS) that represents the health and nutrition status of the non-institutional U.S. civilian population (14, 15). In the present study, data were collected from three NHANES cycles (2009–2010, 2011–2012, and 2013–2014). A total of 30,468 participants were involved in these study periods, with 19,344 completing the *Toxoplasma gondii* IgG analyses. We excluded individuals whose data on three blood heavy metals were missing (*n* = 2,901). Furthermore, we excluded individuals with missing information on covariates (*n* = 5,697). Finally, 10,746 participants were recruited, with 1,869 testing positive for *Toxoplasma gondii* infection. The specific inclusion and exclusion processes are shown in Figure 1.

### Measurement of *Toxoplasma gondii.* IgG levels

The *Toxoplasma gondii.* IgG levels were measured using enzyme immunoassay. The experimental protocol has previously been described in detail (16). The IgG levels were measured in IU/ml and ranged from 0 to 308 IU/mL. The *Toxoplasma gondii.* IgG levels were divided into negative (−) /positive (+) variables (<33 IU/mL, ≥33 IU/mL) (NHANES Analysis Guidelines) (17). Since the skewed distribution of IgG levels cannot be normalized using exponents, logarithms, or contravariant transformations, we did not use IgG concentrations as a continuous variable (3).

### Measurement of heavy metals

From NHANES 2009–2014, the data of three common heavy metals [lead (Pb), cadmium (Cd), and mercury (Hg)] in blood were available. Inductively coupled plasma mass spectrometry (ICP-MS) (PerkinElmer SIMAA 6000) was applied. Detailed experimental protocols and approaches can be found on the NHANES Laboratory Protocol official website.^1^ According to the NHANES criterion, metal concentrations below the limit of detection (LOD) were replaced with the value of LOD divided by the square root of 2 (14).

### Covariates

The covariates corrected in the statistical model included the following variables: age (old people: ≥60 years old, young and middle-aged people: 20–59 years old), sex (male, female), ethnicity (Mexican American, non-Hispanic white, non-Hispanic black, other Hispanic, or other/multiracial), levels of education (<high school, ≥high school), marital status (married or living with partner, widowed or divorced or separated, and never married), body mass index (BMI) (<25, 25–29.90, ≥30 kg/m^2^), family poverty–income ratio (PIR) (≤1.30, 1.31–3.50, >3.50), serum cotinine concentration (>0.011, ≤0.011 ng/mL), and NHANES cycles (18). Serum cotinine concentration has been recognized as a reliable biomarker for objective assessment of smoking and secondhand smoke exposure and is a better measure than self-reported actual smoking dose (19).

### Statistical analysis

Descriptive statistics of the participants’ characteristics and blood metal concentrations were given as mean ± standard deviation (SD), median and interquartile range (IQR), or frequencies (%). Baseline characteristics and blood metal concentrations between *Toxoplasma gondii.* IgG (+) and *Toxoplasma gondii.* IgG (−) participants were compared using a t-test, Mann–Whitney U-test, or χ^2^ test. The concentrations of blood metal were Ln-transformed to approximate normality and further divided into four quartiles (Q1, Q2, Q3, and Q4). We calculated Pearson’s correlation coefficients to pinpoint the correlations between the Ln-transformed blood metal concentrations. All data analyses and processing were carried out by R software (version 4.2.2), and the statistical significance was set as a double-tailed *p*-value of <0.05. Stratified analyses were further conducted based on age (20 ≤ age ≤ 59, age ≥ 60).

First, the weighted multivariate logistic regression was carried out to estimate the association between each heavy metal in blood and *Toxoplasma* infection. The first quartile (Q1) served as the reference, and the results were expressed as odds ratios (ORs) and corresponding 95% confidence intervals (CIs). Age, sex, ethnicity, PIR, marital status, levels of education, BMI, serum cotinine, and NHANES cycles were adjusted in the models to avoid underlying confounders. The dose–reaction relationship between exposure to blood heavy metals and *Toxoplasma* infection was identified using RCS regression performed by the R-packet “rms.” It can not only reflect the linear relationship between heavy metals and *Toxoplasma* infection risk but also uncover the non-linear relationship between the two.

Next, we used two novel and complementary approaches, WQS regression and qgcomp models, to comprehensively investigate the overall effects of three heavy metals in blood on the risk of *Toxoplasma* infection. The WQS model has been described in detail in the past (20). Data were separated into two datasets at random (40% for the training subset and 60% for the validation subset). After bootstrapping 500 times, the R package (“gWQS”) calculated a weighted linear index (ranging from 0 to 1) that represented the total human load of all three metals. The corresponding weight of each metal was equal to its contribution to the WQS index. Considering the uncertainty of the direction of each component in the mixture, we further applied the qgcomp method (21). The model assigned positive and negative weights to each component in the mixture, and the sum of weights in different directions was 1.0.

## Results

### Study population characteristics

A total of 10,746 participants aged 20–80 were incorporated in the analyses, of which 1,869 were *Toxoplasma gondii* IgG (+). The demographic features of all participants are shown in Table 1. It can be found that there were statistical significances in age, sex, race, levels of education, marital status, BMI, and family PIR between *Toxoplasma gondii* IgG (−) and *Toxoplasma gondii* IgG (+) participants (all *p*-values <0.05). People with *Toxoplasma gondii* IgG (+) were more likely to be younger, male, non-Hispanic black, and overweight, with lower education levels and family PIR. Moreover, there was no significant difference between *Toxoplasma gondii* IgG (−) and *Toxoplasma gondii* IgG (+) participants regarding serum cotinine level.

**Table 1:** Characteristics of participants in the NHANES 2009–2014 cycles.

Variable label	*Toxoplasma gondii*. IgG (−) (*n* = 8,877)	*Toxoplasma gondii*. IgG (+) (*n* = 1,869)	Total (*n* = 10,746)	*p*-value
Age, N (%)				
<60	6,289 (70.8%)	1,002 (53.6%)	7,291 (67.8%)	<0.001
≥60	2,588 (29.2%)	867 (46.4%)	3,455 (32.2%)	
Gender, N (%)				
Male	4,219 (47.5%)	1,011 (54.1%)	5,230 (48.7%)	<0.001
Female	4,658 (52.5%)	858 (45.9%)	5,516 (51.3%)	
Race/ethnicity, N (%)				
Mexican American	1,226 (13.8%)	268 (14.3%)	1,494 (13.9%)	<0.001
Other Hispanic	4,278 (48.2%)	735 (39.3%)	5,013 (46.6%)	
Non-Hispanic white	1,685 (19.0%)	367 (19.6%)	2,052 (19.1%)	
Non-Hispanic black	664 (7.5%)	357 (19.1%)	1,021 (9.5%)	
Other Race	1,024 (11.5%)	142 (7.6%)	1,166 (10.9%)	
Educational level, N (%)				
Lower than high school	726 (8.2%)	339 (18.1%)	1,065 (9.9%)	<0.001
Greater than high school	8,151 (91.8%)	1,530 (81.9%)	9,681 (90.1%)	
Marital status, N (%)				
Married/living with partner	5,203 (58.6%)	1,153 (61.7%)	6,356 (59.1%)	<0.001
Widowed/divorced/separated	1921 (21.6%)	489 (26.2%)	2,410 (22.4%)	
Never married	1753 (19.7%)	227 (12.1%)	1980 (18.4%)	
BMI (kg/m^2^), N (%)				
<25	2,717 (30.6%)	461 (24.7%)	3,178 (29.6%)	<0.001
25–29.90	2,846 (32.1%)	669 (35.8%)	3,515 (32.7%)	
≥30	3,314 (37.3%)	739 (39.5%)	4,053 (37.7%)	
Family PIR, N (%)				
≤1.30	2,930 (33.0%)	748 (40.0%)	3,678 (34.2%)	<0.001
1.31–3.50	3,131 (35.3%)	692 (37.0%)	3,823 (35.6%)	
>3.50	2,816 (31.7%)	429 (23.0%)	3,245 (30.2%)	
Serum cotinine, N (%)				
>0.011	2,403 (27.1%)	482 (25.8%)	2,885 (26.8%)	0.256
≤0.011	6,474 (72.9%)	1,387 (74.2%)	7,861 (73.2%)	

Categorical variables were presented as N (%).

BMI, body mass index; N, number of subjects; NHANES, National Health and Nutrition Examination Survey; PIR, poverty income ratio.

### Blood heavy metal concentrations and correlations

**Figure 2:**
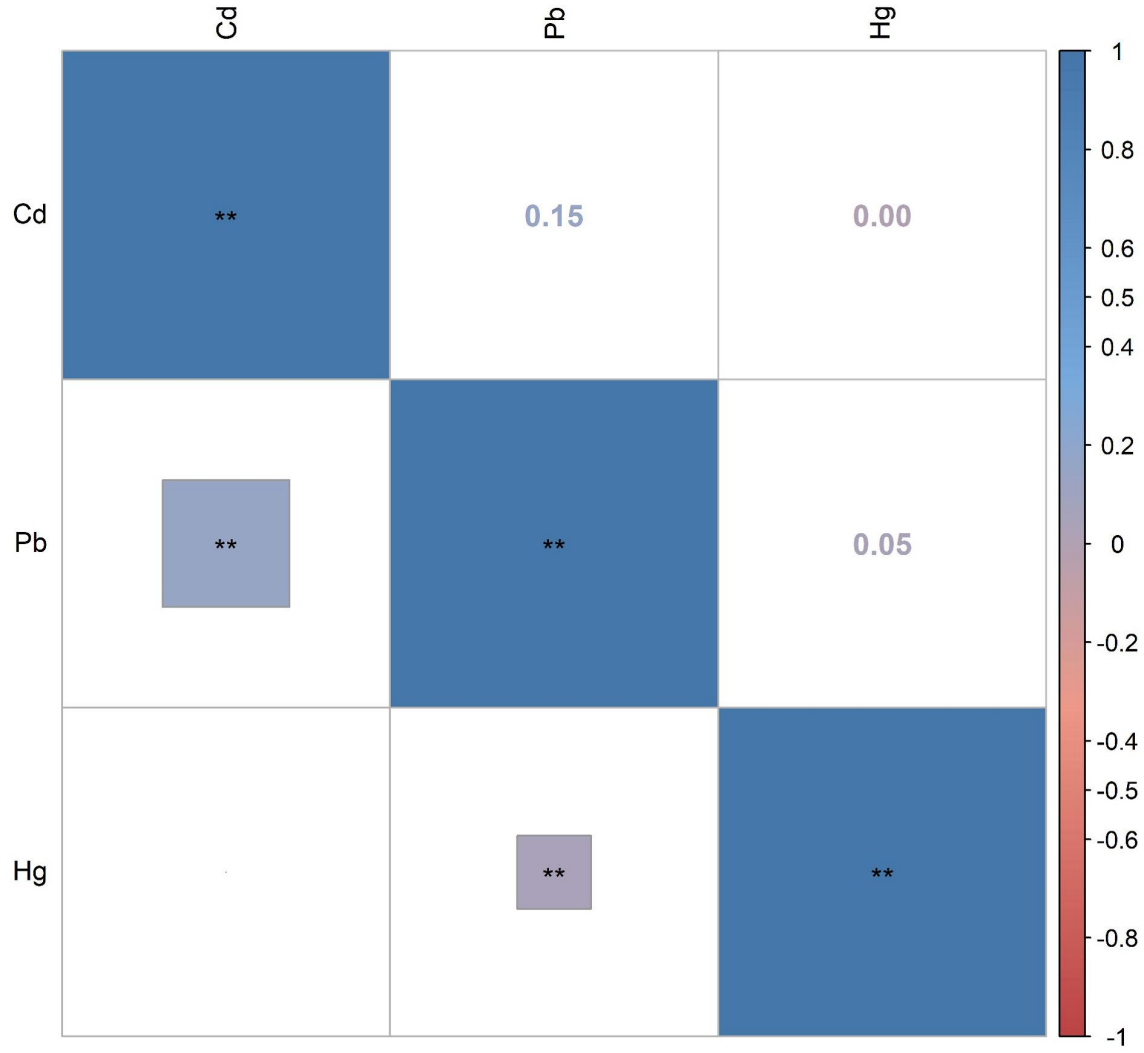
The Pearson correlation between blood metals after In-transformed. *P < 0.05; **P < 0.01.

Supplementary Table S1 presents the distributions and the detection rates of three blood heavy metals. The detection rates of three heavy metals in blood were all more than 85%. The median and IQR of blood Pb, Cd, and Hg were 1.16 (1.08), 0.33 (0.39), and 0.85 (1.30), respectively. Subgroup analyses based on age showed that blood Pb and Hg were significantly higher in older adults than in young and middle-aged populations (all *p*-values <0.001). Figure 2 shows Pearson’s correlation coefficients between these metals after being Ln-transformed. There were relatively weak correlations between blood Pb and Cd (*r* = 0.15), Pb and Hg (*r* = 0.05), and Cd and Hg (*r* = 0.00).

### Logistic regression to analyze the association of single blood heavy metals with the risk of *Toxoplasma gondii* infection

To evaluate the effect of single blood heavy metals on the risk of *Toxoplasma gondii* infection, multivariate logistic regression adjusted for several covariates was conducted, and the results can be found in Table 2. The highest quantile (Q4) of Pb (OR: 2.06, 95%CI: (1.74, 2.45), *p* < 0.001) and Hg (OR: 1.37, 95%CI: (1.18, 1.60), *p* < 0.001) increased the risk of *Toxoplasma gondii* infection compared to the first quantile (Q1). Such significant associations were kept when setting Ln-transformed blood metal concentration as an independent variable [Pb: OR: 1.50, 95%CI: (1.37, 1.63), *p* < 0.001; Hg: OR: 1.13, 95%CI: (1.07, 1.19), *p* < 0.001]. Moreover, the Ln-transformed blood Cd level was also positively correlated with the risk of *Toxoplasma gondii* infection (Cd: OR: 1.08, 95%CI: (1.00, 1.16), *p* = 0.036). Furthermore, subgroup analyses were performed according to age. The positive correlation between blood Pb concentration and *Toxoplasma gondii* infection was statistically significant in both the young and middle-aged group and the older adult group, but more significant in the young and middle-aged group. Moreover, the positive association between blood Hg concentration and *Toxoplasma gondii* infection was significant in the young and middle-aged group but not in the older adult group.

**Table 2:** Associations of single blood metals with Toxoplasma infection risk in the NHANES 2009–2014 cycles.

Blood metal (μg/L)	Continuous		Q1	Q2		Q3		Q4	
	OR (95% CI)	*p*-value		OR (95% CI)	*p*-value	OR (95% CI)	*p*-value	OR (95% CI)	*p*-value
Pb									
Overall	**1.50 (1.37, 1.63)**	**<0.001**	**Ref**	**1.36 (1.15, 1.61)**	**<0.001**	**1.76 (1.49, 2.07)**	**<0.001**	**2.06 (1.74, 2.45)**	**<0.001**
Age < 60	**1.53 (1.38, 1.70)**	**<0.001**	**Ref**	**1.45 (1.16, 1.81)**	**0.001**	**1.86 (1.49, 2.32)**	**<0.001**	**2.60 (2.10, 3.25)**	**<0.001**
Age ≥ 60	**1.20 (1.05, 1.38)**	**0.009**	**Ref**	**1.36 (1.08, 1.70)**	**0.008**	**1.30 (1.03, 1.64)**	**0.025**	**1.36 (1.08, 1.72)**	**0.009**
Cd									
Overall	**1.08 (1.00, 1.16)**	**0.036**	Ref	1.11 (0.95, 1.28)	0.191	**1.16 (1.00, 1.35)**	**0.049**	1.03 (0.88, 1.20)	0.703
Age < 60	1.02 (0.94, 1.11)	0.615	Ref	**1.27 (1.05, 1.54)**	**0.014**	1.07 (0.87, 1.31)	0.535	1.12 (0.91, 1.37)	0.274
Age ≥ 60	1.01 (0.90, 1.14)	0.869	Ref	**1.27 (1.02, 1.58)**	**0.036**	**1.31 (1.04, 1.64)**	**0.021**	1.02 (0.81, 1.29)	0.850
Hg									
Overall	**1.13 (1.07, 1.19)**	**<0.001**	**Ref**	**1.25 (1.08, 1.45)**	**0.003**	**1.28 (1.10, 1.49)**	**0.001**	**1.37 (1.18, 1.60)**	**<0.001**
Age < 60	**1.15 (1.07, 1.24)**	**<0.001**	**Ref**	**1.36 (1.12, 1.67)**	**0.002**	**1.39 (1.13, 1.70)**	**0.001**	**1.56 (1.27, 1.93)**	**<0.001**
Age ≥ 60	1.01 (0.93, 1.10)	0.727	Ref	1.15 (0.92, 1.43)	0.230	1.14 (0.91, 1.43)	0.272	1.13 (0.89, 1.43)	0.324

Models were adjusted for age, sex, race/ethnicity, education levels, poverty income ratio, marital status, body mass index, serum cotinine, and NHANES cycles. Continuous, Ln-transformed concentration of metal; CI, confidence interval; OR, odds ratio; Q, quartile; Ref, reference.

Bold: *p* < 0.05.

### RCS model to analyze the dose–response relationship between single blood heavy metals and the risk of *Toxoplasma gondii* infection

**Figure 3:**
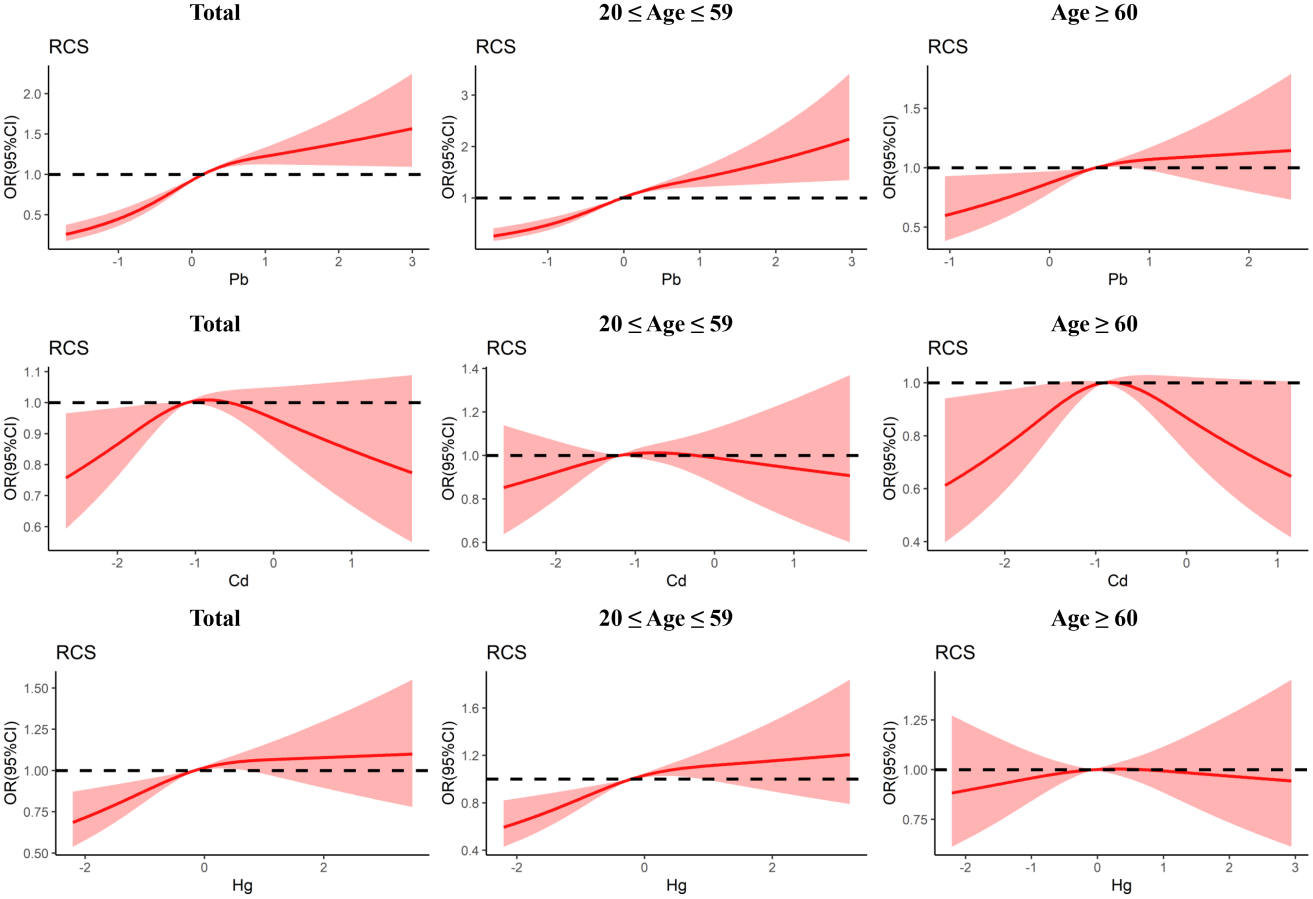
Dose-response relationship between urinary blood metals and Toxoplasma gondii infection were estimated by RCS models in total population and subgroups.

The dose–reaction relationship between three blood heavy metals and the risk of *Toxoplasma gondii* infection estimated using RCS regression is shown in Figure 3. In general, the non-linear and positive association between blood Pb level and *Toxoplasma gondii* infection was observed (*P_nonlinear_* < 0.001, *P_overall_* < 0.001). The risk of *Toxoplasma gondii* infection increased with elevated blood Pb level. The subgroup analyses also indicated a positive association between blood Pb level and *Toxoplasma gondii* infection risk in both the young and middle-aged group and the older adult group, but it was non-linear in the young and middle-aged group (*P_nonlinear_* = 0.003, *P_overall_* < 0.001) and linear in the older adult group (*P_nonlinear_* = 0.223, *P_overall_* = 0.018). The non-linear association between blood Cd level and *Toxoplasma gondii* infection risk in the older adult group (*P_nonlinear_* = 0.013, *P_overall_* = 0.045), but not in the total group and the young and middle-aged group. Moreover, there was a linear and positive association between blood Hg level and *Toxoplasma gondii* infection (*P_nonlinear_* = 0.085, *P_overall_* = 0.001). A similar trend was also identified in the young and middle-aged group (*P_nonlinear_* = 0.095, *P_overall_* < 0.001), but not in the older adult group.

### WQS models to assess the joint effect of co-exposure to three blood heavy metals with the risk of *Toxoplasma gondii* infection

**Figure 4:**
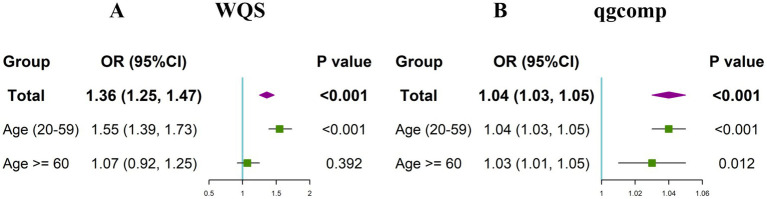
Odds ratios (95% CI) of Toxoplasma gondii infection associated with co-exposure to blood metal mixtures by WQS **(A)** and qgcomp **(B)** analyses.

Next, we further explored the effect of combined exposure to multiple blood heavy metals on the risk of toxoplasma infection. The WQS regression correcting for all covariates was conducted. It can be found that the combined exposure to blood heavy metals was significantly associated with an increased risk of *Toxoplasma gondii* infection (OR: 1.36, 95%CI: (1.25, 1.47), *p* < 0.001) (Figure 4). Among the mixed blood heavy metals, Pb accounted for the highest weight with a value of 0.830, followed by Hg (0.143) and Cd (0.027) (Supplementary Figure S1). Furthermore, subgroup analyses were carried out by age. The results showed that the significant association between co-exposure to blood heavy metals and *Toxoplasma gondii* infection remained in the young and middle-aged group (OR: 1.55, 95%CI: (1.39, 1.73), *p* < 0.001), but not in the older adult group. In the young and middle-aged group, Pb accounted for the highest weight with a value of 0.744, followed by Hg (0.251) and Cd (0.005).

### Qgcomp models to assess the joint effect of co-exposure to three blood heavy metals with the risk of *Toxoplasma gondii* infection

In the qgcomp regression model, after correcting for all confounding factors, we also observed the significant and positive association between co-exposure to blood heavy metals and the risk of *Toxoplasma gondii* infection (OR: 1.04, 95%CI: (1.03, 1.05), *p* < 0.001). The estimated weights of the positive and negative factors are shown in Supplementary Figure S2, where Pb (weighted 0.756) was still the strongest positive driver, followed by Hg (weighted 0.244). The results of subgroup analyses revealed that the significant association between co-exposure to blood heavy metals and *Toxoplasma gondii* infection remained in both the young and middle-aged group (OR: 1.04, 95%CI: (1.03, 1.05), *p* < 0.001) and the older adult group (OR: 1.03, 95%CI: (1.01, 1.05), *p* = 0.012). In the young and middle-aged group, Pb accounted for the highest weight with a value of 0.788, followed by Hg (0.212). In the older adult group, Pb accounted for the highest weight with a value of 0.577, followed by Hg (0.305) and Cd (0.118).

## Discussion

In our cross-sectional analysis of U.S. adults, we applied four diverse statistical techniques, accounting for multiple confounding variables, to thoroughly evaluate the distinct and cumulative influences of three prevalent heavy metals—lead (Pb), cadmium (Cd), and mercury (Hg)—on the risk of *Toxoplasma gondii* infection. The single-metal logistic regression models demonstrated independent correlations between each metal and the risk of infection. Furthermore, restricted cubic spline (RCS) analysis exposed non-linear associations for blood Pb and linear associations for blood Hg with *Toxoplasma gondii* infection. When examining multiple-metal models, both the weighted quantile sum (WQS) and quantile-based g computation (qgcomp) models consistently revealed a positive link between co-exposure to these heavy metals and the risk of *Toxoplasma gondii* infection. Subgroup analyses indicated that this link was particularly pronounced among younger and middle-aged individuals. Notably, the most robust association was observed between lead (Pb) and *Toxoplasma gondii* infection across all statistical methodologies.

Pb is one of the most toxic metals with no biological function (22). Pb is ubiquitous in the environment and may account for 10% of total heavy metal pollution (23). Activities that constitute major sources of lead exposure include industrial activities, mining, lead-glazed ceramic manufacturing, battery recycling, and improper waste disposal (24, 25). There is growing evidence that Pb has multiple adverse health effects, such as cardiovascular disorders, kidney disease, and neurological disorders (26–29). Pb can also enhance the rate of chronic infection by affecting the immune responses. Park et al. found that the blood Pb level was positively correlated with *Helicobacter pylori* infection (30). In a study on the neuroethology of children, Martinez et al. revealed the underlying interaction between Toxoplasmosis prevalence and Pb exposure, which supported the findings of our study (31). Moreover, the non-linear association of blood Pb with *Toxoplasma gondii* infection was observed, which may be related to the relatively long half-life of lead (32). In addition to Pb, the independent association between Cd and Hg with *Toxoplasma gondii* infection was also identified in our study. Cd is a toxic heavy metal that poses a serious threat to human health (33). The rapid development of industry has caused serious Cd pollution (34). Cd can enter the body via a variety of ways, such as the atmosphere, food, water, and soil, and has a half-life of 10–30 years (35). A cross-sectional research using the NHANES data reported that the immune effect of Cd toxicity may be correlated with increased susceptibility to chronic infections (36). Hg is a toxic heavy metal that occurs naturally in the environment in various forms (37). Human activities can release Hg into the air, water, and soil (38). Hg is also released into the environment after it is converted to methylmercury by bacteria. Hg has long been considered a neurotoxicant; nevertheless, recent studies in animals have shown that Hg is also an immunotoxic agent (39). Toxoplasmosis can cause a lifelong infection that can reactivate and arouse severe illness or even death in immunocompromised individuals (40). In short, the influence of these blood heavy metals on toxoplasma infection may be related to their immunotoxicity.

Though the link between exposure to specific metals and *Toxoplasma gondii* infection is established, data on the synergistic effects of metal mixtures remain scarce. In this study, we used weighted quantile sum (WQS) and quantile-based g computation (qgcomp) regression models to assess the collective impact of three prevalent blood heavy metals on the risk of *Toxoplasma gondii* infection. Our findings consistently indicated a positive correlation between exposure to these metal mixtures and the risk of infection. Notably, lead (Pb) was identified as the metal with the highest weighted compound risk by both models. Subsequently, we conducted stratified analyses based on age groups to further explore this association. It can be found that the positive association between co-exposure to blood heavy metal mixtures and *Toxoplasma gondii* infection was stronger in the young and middle-aged group, which may be due to more occupational exposure opportunities to heavy metals in the young and middle-aged population (18).

While the current study utilized a suite of complementary statistical models to explore both the individual and combined influences of blood heavy metals on *Toxoplasma gondii* infection, yielding relatively robust findings, certain inherent limitations should be acknowledged. First, the cross-sectional nature of the survey design precludes any definitive conclusions about causality between heavy metal exposure and *Toxoplasma* infection. Second, the varying half-lives of different metals within the human body complicate the precise measurement of their concentrations. Finally, despite accounting for a range of covariates, not all potential confounding factors could be fully addressed due to data limitations. In conclusion, the relationship between metal mixtures and the risk of *Toxoplasma gondii* infection merits further investigation and validation through prospective, large-scale cohort studies.

## Data Availability

The original contributions presented in the study are included in the article/Supplementary material, further inquiries can be directed to the corresponding author.

## Footnotes

^1^ https://www.cdc.gov/nchs/nhanes/index.htm
