# Supplementary material for: Associations of exposure to blood heavy metal mixtures with Toxoplasma infection among U.S. adults: a cross-sectional study
Source: Front Public Health. 2024 Nov 19;12:1463190. doi: 10.3389/fpubh.2024.1463190 (PMC11611873; doi:10.3389/fpubh.2024.1463190)
Supplement: Supplementary file 1 [file Table_1.DOCX]

**Table S1. The distributions of three blood heavy metals in the NHANES 2009-2014 cycles**

| **Blood metal (μg/L)** | **Detection rates (%)** | **Percentile** | | | | | **IQR** | **Geometric mean** |  | **Mean** | | | |
| --- | --- | --- | --- | --- | --- | --- | --- | --- | --- | --- | --- | --- | --- |
|  |  | **P5** | **P25** | **P50** | **P75** | **P95** |  |  |  | **Total** | **<60** | **≥60** | ***P* value** |
| Pb | 99.7 | 0.41 | 0.75 | 1.16 | 1.83 | 3.70 | 1.08 | 1.19 |  | 1.55 | 1.34 | 1.98 | < 0.001 |
| Cd | 85.4 | 0.11 | 0.21 | 0.33 | 0.60 | 1.60 | 0.39 | 0.36 |  | 0.52 | 0.52 | 0.53 | 0.498 |
| Hg | 90.9 | 0.21 | 0.45 | 0.85 | 1.75 | 5.43 | 1.30 | 0.92 |  | 1.61 | 1.55 | 1.73 | < 0.001 |


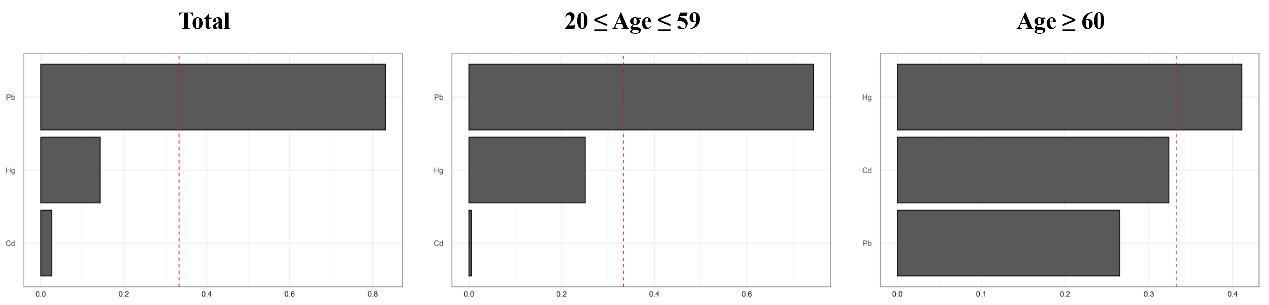


**Fig. S1** Estimated weights of blood heavy metals for *Toxoplasma* infection by WQS models adjusted for age, sex, race/ethnicity, education levels, Poverty Income Ratio, marital status, body mass index, serum cotinine, and NHANES cycles.


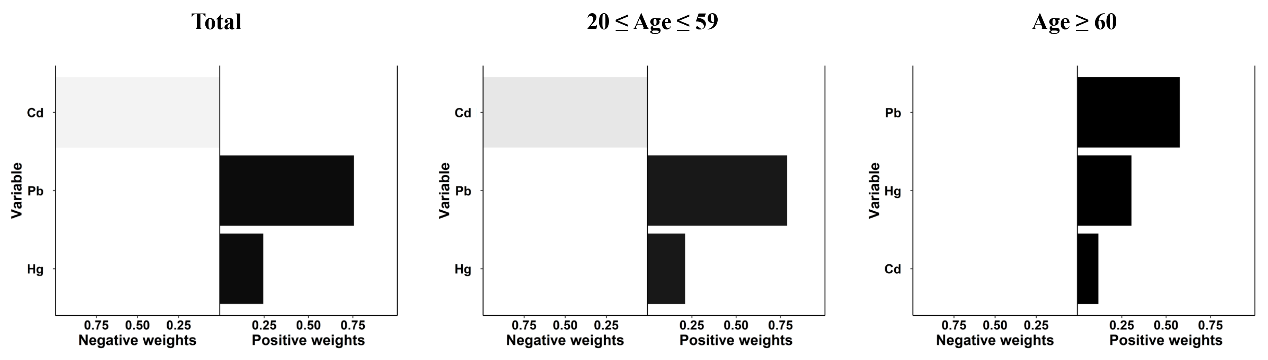


**Fig. S2** Estimated weights of blood heavy metals for *Toxoplasma* infection by qgcomp models adjusted for age, sex, race/ethnicity, education levels, Poverty Income Ratio, marital status, body mass index, serum cotinine, and NHANES cycles.
